# Extracting the latent needs of dementia patients and caregivers from transcribed interviews in japanese: an initial assessment of the availability of morpheme selection as input data with Z-scores in machine learning

**DOI:** 10.1186/s12911-023-02303-3

**Published:** 2023-10-05

**Authors:** Nanae Tanemura, Tsuyoshi Sasaki, Ryotaro Miyamoto, Jin Watanabe, Michihiro Araki, Junko Sato, Tsuyoshi Chiba

**Affiliations:** 1 National Institute of Health and Nutrition, National Institutes of Biomedical Innovation, Health and Nutrition, 3-17 Senriokashinmachi, Settsu, Osaka, 566–0002 Japan; 2 Department of Child Psychiatry and Psychiatry, Chiba University Hospital, Chiba, Japan; 3 Kimura Information Technology Co., Ltd, Saga, Japan; 4 Office of International Programs, Pharmaceuticals and Medical Devices Agency, Tokyo, Japan

**Keywords:** Caregivers, Dementia patients, Latent needs, Machine learning model, Morpheme, Patient public involvement, Z-scores

## Abstract

**Background:**

Given the increasing number of dementia patients worldwide, a new method was developed for machine learning models to identify the ‘latent needs’ of patients and caregivers to facilitate patient/public involvement in societal decision making.

**Methods:**

Japanese transcribed interviews with 53 dementia patients and caregivers were used. A new morpheme selection method using Z-scores was developed to identify trends in describing the latent needs. F-measures with and without the new method were compared using three machine learning models.

**Results:**

The F-measures with the new method were higher for the support vector machine (SVM) (F-measure of 0.81 with the new method and F-measure of 0.79 without the new method for patients) and Naive Bayes (F-measure of 0.69 with the new method and F-measure of 0.67 without the new method for caregivers and F-measure of 0.75 with the new method and F-measure of 0.73 without the new method for patients).

**Conclusion:**

A new scheme based on Z-score adaptation for machine learning models was developed to predict the latent needs of dementia patients and their caregivers by extracting data from interviews in Japanese. However, this study alone cannot be used to assign significance to the adaptation of the new method because of no enough size of sample dataset. Such pre-selection with Z-score adaptation from text data in machine learning models should be considered with more modified suitable methods in the near future.

## Background

 In 2011, 35.6 million dementia patients were reported worldwide, and the number is estimated to double by 2030 and triple by 2050 [1]. In Japan, the number of dementia patients is increasing with the ageing population. In 2020, one-sixth of those aged 65 and above were reported to suffer from dementia [2]. Moreover, more than half of the cases belong to Alzheimer’s type dementia. By 2025, one-fifth of the population aged above 65 is predicted to suffer from dementia [3].

Thus, dementia is a priority public health issue. Moreover, this condition also indirectly affects caregivers, thereby necessitating appropriate support from the health, social, economic, and legal systems [1]. In January 2015, the ‘Comprehensive Strategy to Promote Dementia Policies’ (named the ‘New Orange Plan’ in Japan) was formulated to ensure social dignity for people with dementia [4]. The seven pillars presented in this plan include providing appropriate medical care for people with dementia, developing therapeutic methods, and emphasising the perspectives of dementia patients and their families. However, according to reports in 2014, the scores for treatment satisfaction and drug contribution for Alzheimer’s disease were low at 16.7% and 43.8%, respectively [5]. By contrast, the corresponding scores for hypertension were 89.9% and 95.1%. In other words, developing effective treatments in this therapeutic area is essential. Generally, both patients and caregivers can provide individual insights into health needs. However, dementia treatments often lack the use of effective drugs, which is reflected by the latent health needs of patients.

According to a study, patients and the public must have guaranteed access to medical information, systems, and institutions to ensure patient autonomy in medical care [6]. In Japan, methods have been proposed to ensure that the needs of patients and their caregivers are reflected in drug development. However, these practical methods are yet to be widely adopted by drug developers [7]. Furthermore, the differences in cultural backgrounds and national characteristics between Japan and other countries significantly influence the degree of patient/public involvement in policy making and healthcare. Additionally, Japanese culture is a high-context culture, wherein people can understand each other easily without significant verbal communication. By contrast, western low-context cultures rely on language for communication [8]. The latent needs at this point in time do not mean needs that specifically exist. It is a concept hypothetically defined as some kind of need that patients and caregivers are aware of or unaware of. Patients/caregivers are living with latent needs in addition to actual nee ds, which are not yet concrete in various parts of their lives when they receive medical care. However, latent needs are inconvenient but not sufficiently strongly to be consciously manifested to remain as unknown demands. Consequently, reflecting the latent needs of individuals in policy making and medical care is difficult in the Japanese setting compared with that in other countries. In addition, methodologies or technologies that can identify the latent needs or systems that can translate these needs into policy decisions and health care settings are currently non-existent in Japan.

In recent years, patient narratives have been the focus of attention both in Japan and overseas [9]. A US-based study reported that a machine learning model with random forest could effectively analyse the content of forums and automatically extract information needs [10]. However, Japanese is more complex than English. Thus, further research is required to determine whether the same model can be applied to Japanese text. However, unlike messages written on bulletin boards, transcribed oral interview text generally comprises a variety of lexical expressions and types for each utterance. Furthermore, subjects generally relate to personal experiences in interviews. Therefore, idiosyncratic expressions are expected, in addition to an increase in vocabulary. However, unique expressions may not be effective indicators when predicting latent needs at a national level [11]. Thus, we adopted the Z-score for morphological selection as a new method to eliminate unique expressions and adopt a vocabulary characteristic of latent needs because feature selection as a data pre-processing strategy has proven to be effective and efficient for data mining and machine learning, particularly in the preparation of high-dimensional data. Moreover, studies comparing the applications of Z-score standardisation versus no Z-score standardisation on common machine learning tasks and datasets have been reported [12, 13]. However, studies comparing the application or non-application of Z-score standardisation as a pre-processing step are rare, particularly those dealing with textual data.

This study was an initial assessment of the availability of extracting automatic latent needs from transcribed interviews in Japanese by comparing the accuracy of machine learning models for each adaptation of this new morpheme selection method using Z-scores. The concept of this study involves exploitation of the benefits of low-cost, repeatable, real-time identification of unsatisfied needs to help facilitate patient/public involvement in the medical and social systems.

## Methods

### Data source

This study used the transcribed interviews of dementia patients and caregivers in Japanese from the ‘Health and Illness Narrative’ provided by DIPEx-Japan (https://www.dipex-j.org/dementia/). It included text data from 53 transcribed individual interviews (dementia patients: six women and eight men; caregivers: 29 women and 10 men) recorded between February 2010 and August 2020.

### Data preparation

#### Data annotation

Notably, in this study, a record is defined as a group of sentences provided as an answer by a subject to one question from the interviewer. In the first step, 9647 records were collected and included in the analysis data (patients: 2073 records; caregivers: 7574 records) (Table 1).

**Table 1:** Number of records in the dataset

Position	N	Latent needs	
		Yes	No
All			
Caregiver	7574	343	7231
Patient	2073	63	2010
After sampling			
Caregiver	686	343	343
Patient	126	63	63

**Fig. 1:**
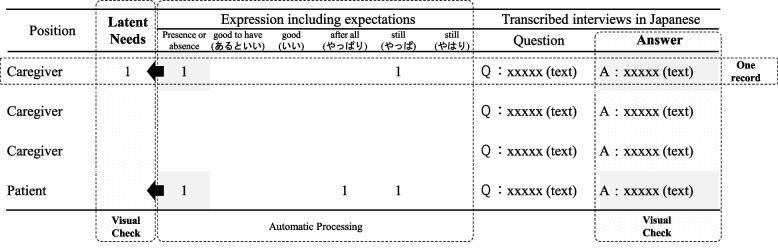
Data annotation

**Fig. 2:**
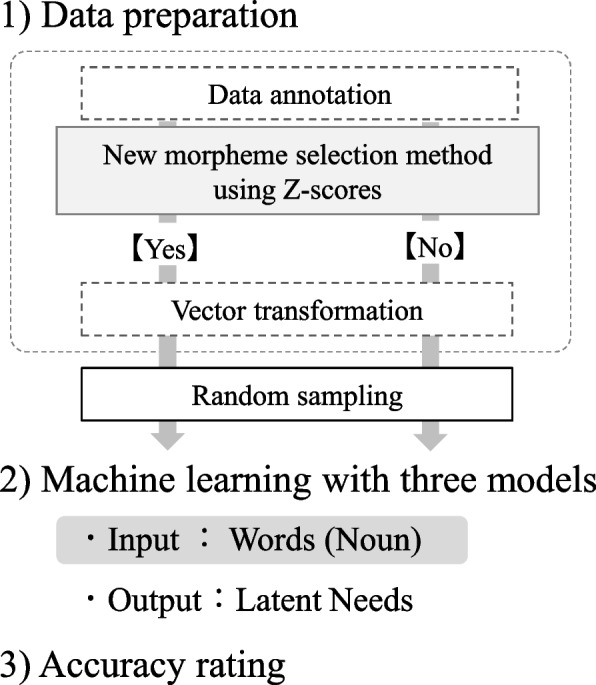
Machine learning models for prediction of latent needs

Subsequently, narratives related to the ‘latent needs’, namely ‘states in which there exists some desire despite a lack of clear self-awareness’, were identified in each record. In particular, the presence or absence of an adverb indicating a certain ‘expectation’ was used as an indicator of a latent need [14]. Accordingly, the expressions ‘it would be good to have / (あるといい in Japanese)’, ‘good / (いい in Japanese)’, ‘after all / (やっぱり in Japanese)’, and ‘still / (やっぱ or やはりin Japanese)’ were considered likely to include the latent needs of the subjects. For example, we defined “A: 皆さまのように、参考になるような話し方ができたらいいんやけど、できない。” as the latent needs, which meant that “I want to talk about something useful for everyone”. The records containing one or more of these adverbs were considered likely to contain the latent needs of the subjects. Finally, two independent researchers tested the presence or absence of the latent needs of the subjects by reading each record as a ‘visual check’. In this step, records expressing differing opinions were discussed by the two researchers until they reached a unanimous verdict on the content. (Figs. 1 and 2)

#### New morpheme selection method using Z-scores

The data in this study were extracted from transcribed interviews in Japanese. Therefore, the records were expected to comprise various peculiar expressions and a few low-occurrence words. Consequently, we predicted that effective machine learning would be difficult if morphemes (words) were used as input data for the automatic extraction of latent needs after morphological analysis.

Therefore, a new method was proposed to select morphemes using Z-scores. MeCab (mecab-python3 0.996.2) was used for the morphological analysis; it analysed the records based on the presence or absence of latent needs. The Z-scores were calculated for each morpheme after counting the number of its occurrences. Note that the Z-score is the difference between the number of occurrences of morpheme ‘x’ and the average number of occurrences of all morphemes, µ, divided by the standard deviation of the number of occurrences of all morphemes, σ. The Z-score of morpheme ‘x’ is calculated using the following formula.

1.1 $$Z - score = \left(x-\mu\right) \sigma$$

Subsequently, a hypothesis was developed focusing on morphemes characterised by the presence or absence of a latent need that would contribute to a higher prediction accuracy in learning. Morphemes for which the magnitude of the difference in Z-scores between records with needs (A) and those without needs (B) was less than 25% of the total were excluded from the data for learning.

The difference in Z-scores was calculated using the following formula:

1.2 $$\surd \left\{\right[(Ax - A\mu )/A\sigma ]- [(Bx -B\mu )/B\sigma \left]\right\}^2$$

This formula was applied to each morpheme to determine whether to select it as an input data for the machine learning model.

Finally, a vector transformation was performed on the selected words (nouns) using the term frequency–inverse document frequency (TF–IDF) method. The TF–IDF is a method for vector expression used in natural language processing. This statistical measure indicates the importance of a word in the target text. It is calculated by multiplying the word frequency value by the inverse of the document frequency value.

#### Random sampling

Notably, the number of records without latent needs significantly outnumbered those with them. Therefore, to implement machine learning appropriately, we used random sampling to ensure an equal number of each type.

#### Comparison of three machine learning models for the prediction of latent needs

The employed hardware was Microsoft Azure (App Service P1V2), and the software was a Jupyter Notebook. The input data were words (nouns), and the output data were latent needs. To select the best machine learning model, three machine learning algorithms were tested, as reported in a similar overseas study [10]: Naive Bayes, support vector machine (SVM), and random forest. We machine-trained each position record separately for training (70%) and test (30%) (Table 2). Standard measures of accuracy, precision, recall, and F-measure were used to analyse the results obtained with and without the new Z-score-based morpheme selection method. (Fig. 2)

**Table 2:** Number of records of in the dataset for machine learning models

Position	N	Dataset	
		Training	Test
Caregiver	686	480	206
Patient	126	88	38

Each record was separated by position for training (70%) and test (30%)

## Results

### Selection of morphemes using Z-scores

Before applying the Z-scores, the caregiver records comprised 3352 words (nouns) with latent needs and 5771 words (nouns) without latent needs; the corresponding numbers for patient records were 1170 words (nouns) and 2271 words (nouns). After applying the Z-scores, the selected caregiver records comprised 1386 words (nouns) with latent needs and 1781 words (nouns) without latent needs; moreover, the selected patient records comprised 468 words (nouns) and 578 words (nouns) with and without latent needs, respectively (Table 3).

**Table 3:** Number of words (Noun) as input data

Latent needs	Z-score	
	No	Yes
Caregiver		
Yes	3352	1386
No	5771	1781
Patient		
Yes	1170	468
No	2271	578

### Random sampling

Random sampling was used to ensure an equal number of records with and without latent needs. Prior to random sampling, the caregiver records consisted of 343 and 7231 records with and without latent needs, respectively; among the patient records, the corresponding numbers were 63 and 2010 records. After random sampling, the selected caregiver records comprised 343 records of each type, and the selected patient records consisted of 63 records of each type (Table 1).

### Machine learning model for the prediction of latent needs

**Fig. 3:**
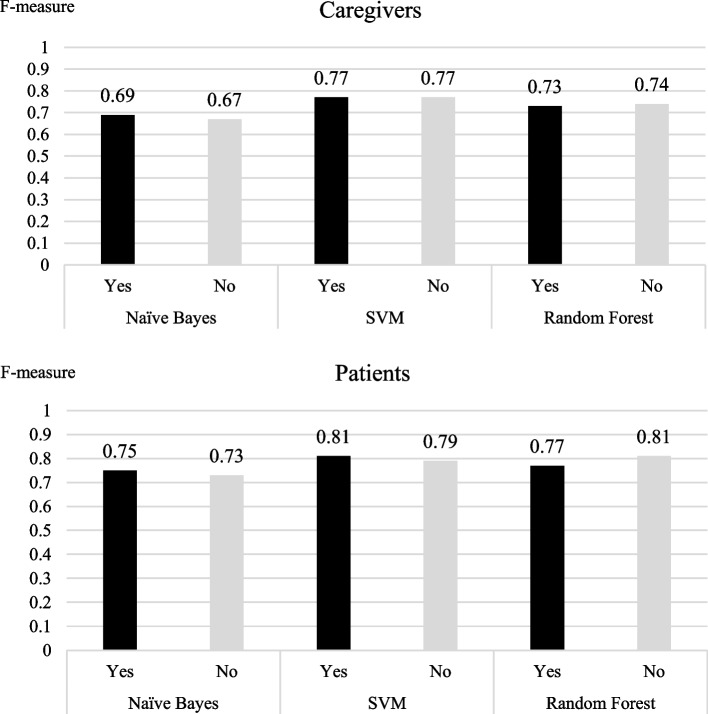
Accuracy in predicting latent needs by new method adaptation. Yes, new method adaptation; No, no new method adaptation

Using the aforementioned standard measures, we analysed the results predicted by the three machine learning models, that is, Naive Bayes, SVM, and random forest, with and without the new morpheme selection method using Z-scores (Fig. 3; Tables 4, 5 and 6).

**Table 4:** Accuracy in predicting latent needs by Naïve Bayes

Position	Standard Measures	Z-score	
		Yes	No
Caregivers	Accuracy	0.75	0.74
	Precision	0.88	0.92
	Recall	0.57	0.53
	**F-measure**	**0.69**	**0.67**
	TN	95	98
	FP	8	5
	FN	44	48
	TP	59	55
Patients	Accuracy	0.79	0.79
	Precision	0.92	1.00
	Recall	0.63	0.58
	**F-measure**	**0.75**	**0.73**
	TN	18	19
	FP	1	0
	FN	7	8
	TP	12	11

*TN* True Negative, *FP* False Positive, *FN* False Negative, *TP* True Positive

**Table 5:** Accuracy in predicting latent needs by SVM

Position	Standard Measures	Z-score	
		Yes	No
Caregivers	Accuracy	0.77	0.79
	Precision	0.77	0.82
	Recall	0.77	0.73
	**F-measure**	**0.77**	**0.77**
	TN	79	87
	FP	24	16
	FN	24	28
	TP	79	75
Patients	Accuracy	0.82	0.82
	Precision	0.83	0.93
	Recall	0.79	0.68
	**F-measure**	**0.81**	**0.79**
	TN	16	18
	FP	3	1
	FN	4	6
	TP	15	13

*TN* True Negative, *FP* False Positive, *FN* False Negative, *TP* True Positive

**Table 6:** Accuracy in predicting latent needs by SVM

Position	Standard Measures	Z-score	
		Yes	No
Caregivers	Accuracy	0.76	0.77
	Precision	0.84	0.87
	Recall	0.65	0.64
	**F-measure**	**0.73**	**0.74**
	TN	90	93
	FP	13	10
	FN	36	37
	TP	67	66
Patients	Accuracy	0.76	0.84
	Precision	0.75	1.00
	Recall	0.79	0.68
	**F-measure**	**0.77**	**0.81**
	TN	14	19
	FP	5	0
	FN	4	6
	TP	15	13

*TN* True Negative, *FP* False Positive, *FN* False Negative, *TP* True Positive

Among the caregiver records, SVM was the most accurate both with and without the new morpheme selection method using Z-scores. The F-measure values for the SVM, random forest, and naive bayes models were 0.77, 0.74, and 0.67 before applying the Z-scores and 0.77, 0.73, and 0.69 after applying the Z-scores, respectively.

For the patient records, SVM was the most accurate when paired with the new morpheme selection method using Z-scores, whereas random forest was the most accurate without the new morpheme selection method using Z-scores. The F-measure values for the SVM, random forest, and naive bayes models were 0.79, 0.81, and 0.73 before applying the Z-scores and 0.81, 0.77, and 0.75 after applying the Z-scores, respectively.

## Discussion

Machine learning models were developed to automatically extract the latent needs of patients and their caregivers from transcribed interviews in Japanese. The Naive Bayes or SVM model paired with the new method exhibited a higher prediction accuracy (F-values) for both caregiver and patient records; this was particularly evident for the SVM with adequate prediction levels. This indicates that using the new morpheme selection method using Z-scores as a pre-processing step, a higher prediction level could be secured while appropriately reducing information.

For example, a study conducted in the US revealed that Z-scores could be used with the SVM to successfully identify important keywords in human genetics-related articles with greater accuracy compared to all other articles [15]. Regardless of the size of the dataset, the selection of important keywords using the Z-score contributes to the selection of appropriate feature parameters for the SVM [16]. Consequently, the SVM was able to classify text classes properly. Therefore, it exhibited the highest accuracy even when using the Z-score. Conversely, Naive Bayes functions on the assumption that the data features are independent and mutually uncorrelated. Therefore, considering that this assumption is unrealistic, the SVM was considered a better choice for prediction. However, the adaptation of the new method resulted in a lower prediction accuracy in the case of random forest. This is because random forest builds a machine learning model by considering the interaction of features. Therefore, the prediction accuracy may be reduced if the features selected in pre-processing do not adequately capture the interaction.

### Strengths and limitations

This is the first study to compare the accuracy of a machine learning model by adopting Z-scores to predict the latent needs of patients or their caregivers using transcribed interviews in Japanese as a data source. However, there are three study limitations. At first, this cannot be adapted directly to all disease domains, as the characteristics of words vary depending on the disease owing to the diversity of symptoms or severity of disease in the Japanese language. This study was an initial assessment, so we consider the evaluation of differences in latent needs by topic classification due to differences in positions to be the next step in the research. Finally, the dataset in this study was small size, so future consideration is needed for further evaluation of the new methods.

## Conclusion

A new scheme for machine learning models was developed to predict the latent needs of dementia patients and their caregivers by extracting related data from interviews in Japanese. Z-score adaptation with SVM in dementia patients showed the enough prediction accuracy of machine learning models. However, this study alone cannot be used to assign significance to the adaptation of the new method because of no enough size of dataset. Such pre-selection with Z-score adaptation from text data in machine learning models should be considered with more modified suitable methods in the near future.

## Data Availability

The data that support the findings of this study are available from DIPEx-Japan (https://www.dipex-j.org/dementia/), but restrictions apply to the availability of these data, which were used under license for the current stud and are hence not publicly available. Therefore, the authors confirm that the data supporting the findings of this study are available within the article.
